# Detection of ovarian cancer using plasma cell-free DNA methylomes

**DOI:** 10.1186/s13148-022-01285-9

**Published:** 2022-06-09

**Authors:** Huaiwu Lu, Yunyun Liu, Jingyu Wang, Shaliu Fu, Lingping Wang, Chunxian Huang, Jing Li, Lingling Xie, Dongyan Wang, Dan Li, Hui Zhou, Qunxian Rao

**Affiliations:** 1 Sun Yat-Sen Memorial Hospital of Sun Yat-sen University, Guangzhou, China; 2 Shanghai Danbei Medical Technology Co., Ltd, Shanghai, China

**Keywords:** Ovarian cancer, cfDNA methylation, Biomarkers, Early cancer detection, cfMeDIP-seq

## Abstract

**Background:**

Ovarian cancer (OC) is a highly lethal gynecologic cancer, and it is hard to diagnose at an early stage. Clinically, there are no ovarian cancer-specific markers for early detection. Here, we demonstrate the use of cell-free DNA (cfDNA) methylomes to detect ovarian cancer, especially the early-stage OC.

**Experimental design:**

Plasma from 74 epithelial ovarian cancer patients, 86 healthy volunteers, and 20 patients with benign pelvic masses was collected. The cfDNA methylomes of these samples were generated by cell-free methylated DNA immunoprecipitation and high-throughput sequencing (cfMeDIP-seq). The differentially methylated regions (DMRs) were identified by the contrasts between tumor and non-tumor groups, and the discrimination performance was evaluated with the iterative training and testing method.

**Results:**

The DMRs identified for cfDNA methylomes can well discriminate tumor groups and non-tumor groups (ROC values from 0.86 to 0.98). The late-stage top 300 DMRs are more late-stage-specific and failed to detect early-stage OC. However, the early-stage markers have the potential to discriminate all-stage OCs from non-tumor samples.

**Conclusions:**

This study demonstrates that cfDNA methylomes generated with cfMeDIP-seq could be used to identify OC-specific biomarkers for OC, especially early OC detection. To detect early-stage OC, the biomarkers should be directly identified from early OC plasma samples rather than mix-stage ones. Further exploration of DMRs from a k larger early-stage OC cohort is warranted.

**Supplementary Information:**

The online version contains supplementary material available at 10.1186/s13148-022-01285-9.

## Background

Ovarian cancer (OC) is the most lethal gynecologic cancer for its low five-year survival rate of 46% [1, 2]. Over the decades, even though the treatment of ovarian cancer has been evolved, there is not much improvement in the survival rate [3]. One main reason is about three-quarters of ovarian cancer patients were diagnosed at late stage (stage III–IV) for the lack of obvious early symptoms [1, 4]. The late diagnosis makes it surgically challenging to reach the complete resection, the only independent factor which could significantly impact the overall survival rate [5]. Early detection of ovarian cancer can be a remarkable strategy to improve this situation as the five-year survival rate of early-stage ovarian cancer can reach 92% [1]. Moreover, the surgery for early-stage ovarian cancer will be much simpler and will allow at least partial patients to preserve their fertility and to have a better quality of life [6, 7].

Several screen trials were designed for the early detection of ovarian cancer. In the UKCTOCS trial, postmenopausal women randomly received CA125 testing or annual transvaginal ultrasound [8]. In the PLCO trial, women underwent both CA125 testing and transvaginal ultrasound [9]. These trials show no significant mortality reduction but documented harms [8, 10, 11]. Thus, the screening in average-risk women with the notable clinical marker CA125 is not commended for the early detection of ovarian cancer. Other serum proteins were also discussed in ovarian cancer detection, including HE4, CEA, osteopontin, etc. [12, 13]. As these protein markers are also elevated in benign conditions, their performance requires further studies (Table 1).

**Table 1:** Patient characteristics

Characteristics	Malignant	Healthy	Benign
Age			
Median (range)	53 (29–77)	53 (42–67)	41 (26–67)
CA125			
Median (range)	308 (6.5–16,608)	13.49 (4.21–31.70)	47.65 (10.5–183.4)
Tumor histology			
High-grade serous	52		
Endometrioid	6		
Clear cell	12		
Mucinous	3		
Mixed	1		
FIGO stage			
I	19		
II	9		
III	43		
IV	3		

cfDNA shed into the bloodstream is quite informative, and the load and the genetic pattern of tumor correlated cfDNA are widely used as biomarkers for cancer monitor and therapy [14, 15]. Recently, cfDNA methylation shows a promising role in the early detection of cancer. DNA methylation alteration is one of the earliest events during carcinogenesis, which represses tumor suppression genes, activates tumor oncogenes, and promotes cancer transformation [16–18]. The alterations of DNA methylation can be detected in the blood even with a very low abundance of circulating tumor DNA (ctDNA), which would be sensitive for cancer detection at the early cancer stage or even before clinical diagnosis [19–21]. Several cfDNA methylation markers have already been discussed in ovarian cancer [22]. However, for the lack of the genome-wide methylation information of cfDNA, their early detection capacity is limited [20]. Lately, cfMeDIP-seq shows its ability to detect low-abundance methylation alterations in several cancer types, including renal cancer, lung cancer, pancreatic cancer, and glioma [21, 23], demonstrating its potential in early-stage cancer detection [21, 24]. In this study, we generated cfDNA methylomes for both early and late-stage ovarian cancer by cfMeDIP-seq. We found the DMRs identified in OC groups could discriminate them from non-tumor samples. Further, we found that the DMRs identified from late-stage OC samples show a late-stage specific pattern and not suitable for the detection of early-stage OC. The majority of cfDNA methylation markers of early-stage OC are also more significantly altered in the early stage. Still, the whole set of early-stage markers could be used for the detection of all-stage of OC.

## Methods

### Patient plasma samples

Plasma samples presented in this study were collected from Sun Yat-Sen Memorial Hospital upon approval of institutional ethics committees (SYSEC-KY-KS-2021-084). All participants provided written informed consent. Seventy-four plasma samples were collected preoperatively from epithelial ovarian cancer patients without any pretreatment. The stage of cancer was surgically evaluated according to FIGO stage guidelines. In addition, 86 plasma samples from healthy volunteers with serum CA125 lower than 35 IU/mL and 20 samples from patients with benign pelvic masses were selected as controls.

### Sample processing and cfDNA extraction

5–10 mL peripheral blood was collected in Streck cell-free DNA tubes, and plasma was isolated within 48 h, frozen at − 80 °C. cfDNA was extracted from 2 to 4 mL plasma using Qiagen Circulating Nucleic Acids Kit (Qiagen), according to manufacturer’s instructions, measured with Qubit fluorometer (Life Technologies), and stored at − 80 °C.

### cfMeDIP-seq protocol

10 ng cfDNA was used for Library preparation with Kapa HyperPrep Kit (Kapa Biosystems) and cfMeDIP with Diagenode MagMeDIP kit (catalog no. C02010021) according to the previously published protocols [21, 25]. The library was amplified with KAPA HiFi Hotstart ReadyMix (KAPA Biosystems) and NEBNext Multiplex Oligos for Illumina (New England BioLabs) as follows: initial denaturation at 95 °C for 3 min, followed by 14–15 cycles of 98 °C for 20 s, 65 °C for 15 s, 72 °C for 30 s, and the final extension at 72 °C for 1 min. After purification with Beckman Agencourt AMPure XP beads, amplified libraries were measured with Qubit fluorometer (Life Technologies) and Bioanalyzer 2100 (Agilent) and then pooled and sequenced (Wuxi NextCode) on an Illumina NovaSeq 6000 system to generate 150 bp paired-end reads.

### cfMeDIP-seq data processing

After sequencing, the sequenced reads were aligned to the hg19 genome using Bowtie [26] with the default settings. The generated SAM files from hg19 alignment were converted to BAM format, ensuring the removal of duplicate reads, and the reads were then sorted and indexed using SAMtools [27] before subsequent analysis with the R package MeDIPS [28]. The CpG enrichment score, as a quality control measure for the immunoprecipitation reaction, was also calculated with the MeDIPS package [28].

### Machine learning approaches for in-group samples classification

Data from cfMeDIP-seq profiles were first reduced to map functional regions including CpG islands, shores, shelves, FANTOM5 human enhancers, and promoters as previously described [29, 30]. For all analyses, models were generated exclusively using samples in the training cohort, and model performance was tested in held-out samples (samples in the test sets). For each two-group comparison, samples were partitioned into 100 independent training and testing cohorts in an 80–20% manner. 100 glmnet models (tumor versus non-tumor) were developed with the top 300 DMRs identified using DESeq2 [31] to estimate the probability of a sample being a tumor sample with the R package caret [32]. Model performance was evaluated with held-out samples in test cohorts by computing the area under the receiver operating characteristic (ROC) curve (AUROC).

To determine whether DMRs identified from late-stage OC samples and healthy samples could distinguish early-stage samples from non-tumor samples, we first performed the differential analysis with 46 late-stage OC samples and 50 healthy samples random sampling from a 86 healthy sample cohort by DESeq2 [31]. Then, a glmnet model was developed using the top 300 DMRs from late-stage OC and selected healthy samples with normalized CPM (count per million reads) value. Finally, model performance was evaluated with early-stage samples and held-out non-tumor samples by computing the AUROC. To evaluate whether early-stage specific DMRs could classify late-stage OC samples with non-tumor samples effectively, we generated similar models using DMRs between early-stage OC samples and randomly sampled healthy samples and evaluated model performance in a similar way.

## Results

### Performance of cfMeDIP-seq data in ovarian cancer and non-cancer samples

To identify ovarian cancer-specific cfDNA DMRs, we used a published cfMeDIP-seq protocol which can sensitively detect as low as 0.001% tumor DNA from a 10 ng DNA mixture [21]. We performed cfMeDIP-seq on 190 plasma samples, collected from 74 epithelial ovarian cancer patients (*n* = 28 stage I–II, *n* = 46 stage III–IV), 86 age-matched healthy volunteers, and 20 benign controls. Following the standard cfMeDIP-seq data processing protocol, the mapping rates of all samples range from 87 to 94%, and these reads were highly enriched in CpG island regions (Additional file 1: Fig. S1A; Additional file 2: Table S1). We further used the CpG enrichment score [28] to evaluate the CpG enrichment profiles. The mean CpG enrichment score is between 3.33 and 3.60 in all sample groups (Additional file 1: Fig. S1B), similar to the results in the previous cfMeDIP-seq papers [21, 23].

### Classification of ovarian tumor samples using cfDNA methylomes

**Fig. 1:**
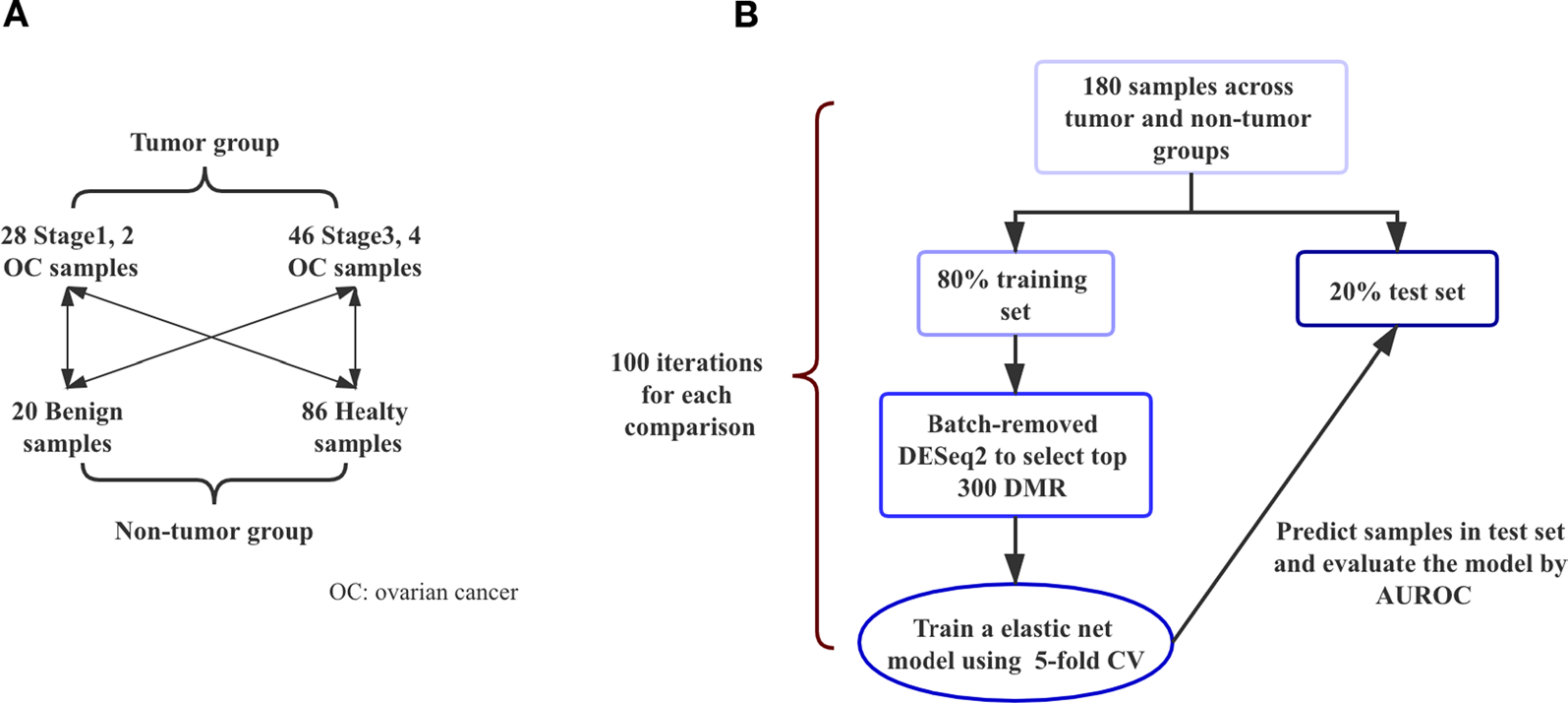
Design of ovarian cfMeDIP-seq dataset analysis. (**A**) Design of tumor and non-tumor cohorts. Classification analyses were performed between two groups linked by a double-headed arrow. (**B**) Flowchart of machine learning algorithm used to train and evaluate cfMeDIP profiles in the detection and classification of ovarian cancer

**Fig. 2:**
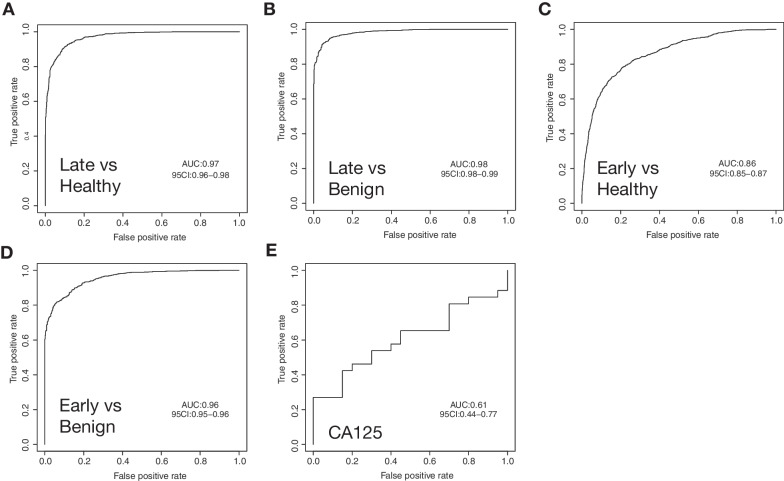
Discriminations of ovarian tumor and non-tumor groups with cfDNA methylomes. (**A**)–(**D**) ROC curves for classifiers generated from 100 iterations of training sets comparing A. late-stage ovarian cancer (OC) versus healthy group, (**B**) late-stage OC versus benign group, (**C**) early-stage OC versus healthy group, (**D**) early-stage OC versus benign group. (**E**) The ROC curve of the discrimination between early-stage OC and benign samples by CA125

To test whether cfMeDIP-seq profiles could discriminate cancer and non-cancer samples, we made contrasts with every two groups (Fig. 1A) and evaluated the ability of differential methylated regions (DMRs) for sample classification in each contrast. We first test the performance of cfMeDIP-seq in groups of late-stage ovarian cancer and healthy samples. Following the previously published method [21, 24], we evaluated the performance of discrimination with the iterative training and testing method. 80% of late-stage samples and healthy controls were randomly selected for each iteration as a training set for DMR identifications. Top 300 DMRs were used to perform elastic-net logistic regression with a glmnet model [32]. And the classification model was used to calculate the sample methylation scores for the held-out 20% samples (Fig. 1B). This training–testing procedure was repeated 100 times, and the discrimination power was assessed with all the predictions of held-out samples during the whole iterations and presented by the area under the receiver operating characteristic curve (AUROC). Across 100 training–testing sets, late-stage OC samples were assigned a higher median methylation score than all healthy samples (Fig. S2B), with acuminating AUROC of 0.97 (95% confidence interval (CI) 0.96–0.98; Fig. 2A). To evaluate the classification performance of the top 300 DMRS identified with cfMeDIP-seq between late-stage samples and benign samples, we conducted a similar analysis and also found a high classification performance by methylation score (Additional file 1: Fig. S2B), with a cumulative AUROC of 0.98 (95% CI 0.98–0.99; Fig. 2B). It demonstrated that DMRs identified with cfMeDIP-seq can classify late-stage OC from non-tumor samples.

To further assess the capacity of cfMeDIP-seq in early-stage detection of ovarian cancer, the same analysis was performed in early-stage OC versus non-tumor controls. Even with a limited number of early-stage samples, we observed a promising classification performance in early-stage OC versus healthy controls (AUROC = 0.86, 95% CI 0.85–0.87; Fig. 2C), and also in early-stage OC versus benign controls (AUROC = 0.96, 95% CI 0.95–0.95; Fig. 2D). Here, we also tested the discrimination power of the widely used OC protein marker CA125 in early-stage OC versus benign samples and observed a poor performance (Fig. 2E). By contrast, the tumor-specific DNA methylation markers from cfMeDIP-seq profiles showed much higher classification performance, revealing its potential in the early detection of ovarian cancer.

### Performance of late-stage methylation markers in early-stage samples

**Fig. 3:**
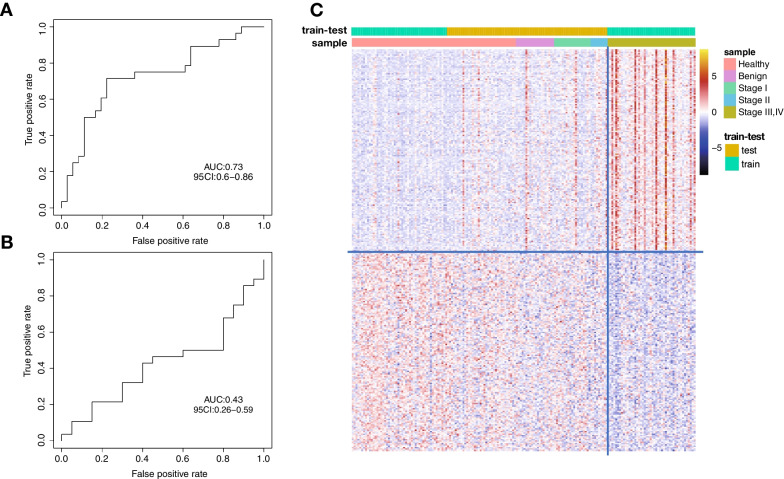
Classification of early-stage ovarian cancer using DMRs generated from late-stage OC and healthy group. (**A**) Heatmap of the top 300 DMRs between late-stage OC and healthy group. (**B**) ROC curve of the classification between early-stage OC and healthy group by the DMRs identified from late-stage OC versus healthy samples. (**C**) ROC curve of the classification of early-stage OC/benign samples by the late-stage OC/healthy DMRs

As it is difficult to obtain a large amount of early-stage OC samples, we wondered whether the ovarian cancer-specific markers identified from late-stage cancer samples could be used to detect early ovarian cancer samples. A glmnet model was trained with the methylation profiles of 46 late-stage cancer samples and 50 healthy control samples using the top 300 DMRs between the two groups to test this assumption. The trained model's performance was first evaluated on the early-stage OC samples and 36 held-out healthy controls, and we observed a comprised discrimination performance (AUC = 0.73, 95% CI 0.6–0.86; Fig. 3A, B). The discrimination ability of this model was weaker than the one generated with tumor-specific markers identified in early-stage samples versus healthy controls markers and would be limited for clinical applications considering the low incidence of ovarian cancer. We further test the performance of this model to discriminate early-stage samples and benign samples and found no classification between these two groups (AUC = 0.45, 95% CI 0.26–0.59; Fig. 3C).

To further understand the characteristic of these OC markers identified from late-stage samples, we checked the methylation levels of these markers in all samples. These markers indeed show a significantly hyper-/hypomethylated pattern in late-stage samples, but these differentially methylated patterns were compromised or lost in early-stage OC samples (Fig. 3A). These observations indicate that the methylation markers identified from late-stage OC cannot be used directly to detect early-stage OC.

### Performance of early-stage methylation markers in late-stage samples

**Fig. 4:**
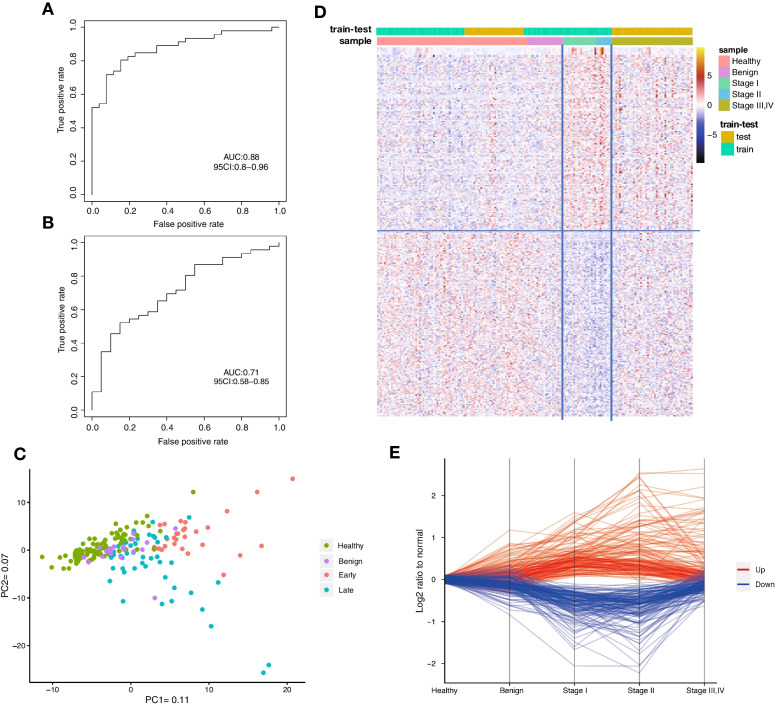
Classification of late-stage ovarian cancer using DMR from early-stage OC and healthy group. (**A**) ROC curve of early-stage OC/healthy DMRs in late-stage OC/healthy classification. (**B**) ROC curve of early-stage OC/healthy DMRs in late-stage OC/Benign classification. (**C**) Heatmap of the top 300 DMRs between early-stage OC and healthy group. (**D**) The top 300 DMRs between early-stage OC and the healthy group were used to generate principal component (PC) plots for four cohorts. (**E**) Relative methylation alteration of the top 300 DMRs identified in early-stage OC/healthy samples was calculated in all sample groups. Hypermethylation was present in the red line, and hypomethylation in blue

Some tumor-specific methylations were reported to show a sequential methylation alteration pattern during the cancer progression [33, 34]. A similar accumulative DNA methylation pattern was also found in ovarian tumor tissues and gastric tumors [35, 36], revealing that some early-stage tumor methylation markers have a consistent or progressed pattern and could be used to detect both early- and late-stage tumors. To test whether the OC methylation markers we identified in early-stage samples could also be used to detect the late-stage OC, we then evaluated the classification efficiency of early-stage markers in late-stage samples. Similarly, we built a glmnet model with the top 300 DMRs between 28 early-stage samples and 50 healthy controls and evaluated its performance in late-stage samples. The early-stage model showed a satisfactory discrimination capacity to classify late-stage samples and healthy samples (AUC = 0.88, 95% CI 0.8–0.96; Fig. 4A), and a relatively weaker capacity in discrimination of late-stage cancer samples and benign controls (AUC = 0.71, 95% CI 0.58–0.85; Fig. 4B). These results confirmed that the early-stage markers could generally present the molecular characteristics of ovarian cancer in all stages. A similar result was also observed when we visualized 4 groups of samples with early-stage OC markers by the top 2 principal components from principal component analysis (PCA). The early-stage markers could separate different groups, although the discriminative ability was lightly decreased between late-stage OC and benign samples (Fig. 4C).

We further evaluated the methylation alterations of these early-stage markers among all four group samples (Fig. 4D, E). Contrary to our expectation, a fraction of early-stage markers showed continuous alteration tendency from healthy to stage II samples, but with less differentially methylated levels in stage III/IV samples. Nearly, all the hypomethylated DMRs reverse to the basal level in the late-stage samples. Depending on whether the DMRs reverse their hypomethylation pattern at stage I or stage II, the hypomethylated DMRs can be clearly divided into two groups. A similar reversal pattern is also observed in hypermethylated DMRs. Still, about a quarter of hypermethylated DMRs maintain or increase their methylated levels in late-stage plasma, which may contribute to the discrimination of late-stage cancer.

## Discussion

DNA methylation is increasingly involved in the research and application of cancer early detection. In precursor lesions of colorectal cancer, a cancer-similar accumulation of DNA methylation can already be observed [37]. Several DNA methylation markers can be detected two years before the clinical diagnosis of ovarian cancer [20]. A recent study showed that cancer detection by a group of DNA methylation markers can be up to four years before the conventional diagnosis [38]. All these works confirmed the potential of DNA methylation markers in the early detection of cancer. cfMeDIP-seq provides a cost-efficient way to generate genome-wide DNA methylation profiles directly from plasma cfDNA. This technique has been proved for its discriminative capacity in many carcinomas, including intracranial tumors [21, 23, 24]. Here, the first time, we generated the genome-wide cfDNA methylomes of ovarian cancer patients along with benign and healthy controls with cfMeDIP-seq. Within each contrast of tumor and non-tumor group, the classifier developed from methylation profiles can effectively discriminate every two groups (Fig. 2A–D), demonstrating the capacity of cfMeDIP-seq to identify the cfDNA methylation alterations in ovarian cancer. Comparing with the performance of the widely used clinical marker CA125 which elevates in fewer than 50% of early-stage OC cases and also elevates in some benign conditions [39, 40], cfDNA methylation markers show more potential to identify early OC patients from the average-risk population. But further works are required.

As there are no apparent symptoms, early-stage OC patients are usually diagnosed incidentally [41]. It will take a very prolonged period of time to collect enough early-stage OC samples for tumor marker identification and validation. As described in a previous study, one solution to solve this common challenge was using DNA methylation markers identified from the OC cohort mainly consisted of late-stage samples for the early detection of ovarian cancer [20]. Although it is quite common to use stage-mixed tumor samples as a discovery set to identify markers for the detection of both early- and late-stage cancer, the performance of these markers usually declined in the early-stage samples [19, 20, 42]. In an extreme case, we evaluated the performance of the methylation markers of late-stage OC versus healthy controls in early-stage samples. The late-stage markers show a distinctive late-stage-specific pattern in plasma, which would limit their contribution to the detection of early-stage ovarian cancer. We also compared the top 300 late-stage markers with the top early-stage markers, and only four of them are overlapped (data not shown). This observation confirms the limited capacity of plasma cfDNA methylation markers from late-stage ovarian cancer samples for early-stage ovarian cancer detection.

Although the late-stage cfDNA methylation markers could not be applied in early-stage detection, these cfDNA markers reflected a sequential pattern during ovarian cancer progression, consistent with previously described in ovarian cancer tissues [43, 44]. However, a large proportion of hypermethylated markers and all hypomethylated markers identified in the early-stage plasma are not altered according to cancer progression. These different cfDNA methylation patterns are likely due to either the complexity of plasma as a highly different proportion of ctDNA in plasma between stages [14, 45] or the possible diverse DNA methylation patterns during cancer progression. Though more evidence is required, the non-sequential methylation pattern has been discovered in the development of cutaneous squamous cell carcinomas [46]. In either of the possibility described above, it has to consider the optimal proportion of different stages of samples in the biomarker discovery cohort to achieve the best specificity for all stages. Considering the low incidence of ovarian cancer and the clinical requirement for detecting early-stage patients [4, 47], the optimal markers should be generated directly from early-stage samples to achieve high specificity.

In this study, with the limited number of early-stage OC samples, we cannot generate a set of optimized markers from a training group of early-stage OC and test them in an independent cohort. Collection of more early-stage samples and further work will be needed to test these cfDNA methylation markers both in the patients with unclear pelvic masses and in an average-risk population to evaluate their discrimination capacity more precisely.

## Conclusions

In summary, with cfDNA methylomes generated by cfMeDIP-seq, we can discriminate OC patients, especially early-stage OC patients with health and benign controls. We observed that the top OC cfDNA methylation markers have a stage-specific pattern, indicating that it is necessary to identify early-stage OC markers directly from an early-stage cohort rather than a mix-stage one.

### Supplementary Information

**Additional file 1.** Supplementary figures of the article.

**Additional file 2.** Supplementary tables of the article.

## Data Availability

All data generated or analyzed during this study are included in this published article and its supplementary information files.
