# Supplementary material for: Detection of ovarian cancer using plasma cell-free DNA methylomes
Source: Clin Epigenetics. 2022 Jun 9;14:74. doi: 10.1186/s13148-022-01285-9 (PMC9185905; doi:10.1186/s13148-022-01285-9)
Supplement: Supplementary file 1 — Additional file 1. Supplementary figures of the article. [file 13148_2022_1285_MOESM1_ESM.docx]

**Supplementary figures**

**
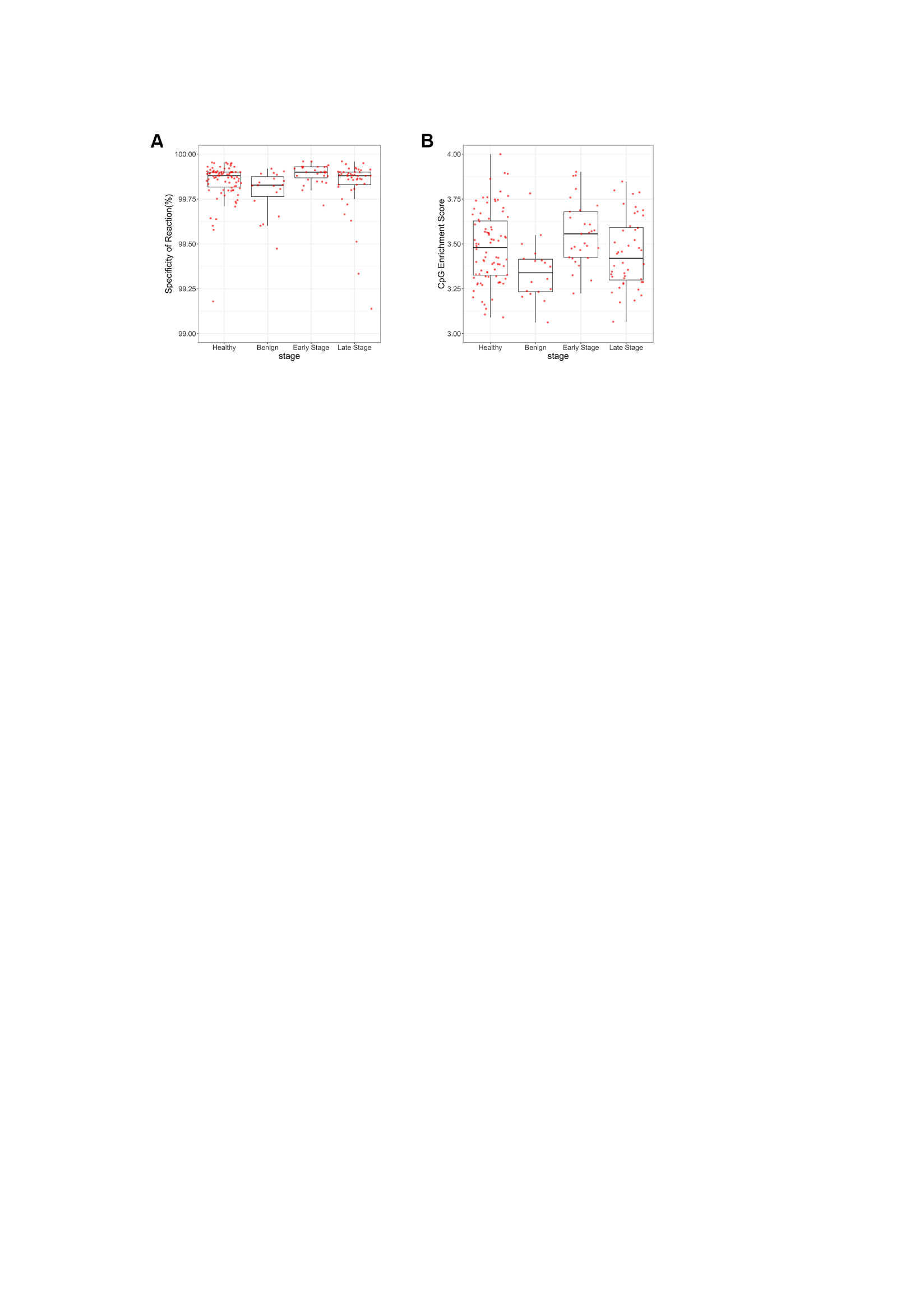
**

**Fig S1. Statistics of cfMeDIP-seq dataset quality assessment.** A. Specificity of reaction rate in four groups. The specificity of the reaction was calculated using methylated and unmethylated spiked-in *A. thaliana* DNA. B. Boxplot of CpG enrichment score in four groups. CpG enrichment score is calculated by the MEDIPS package.


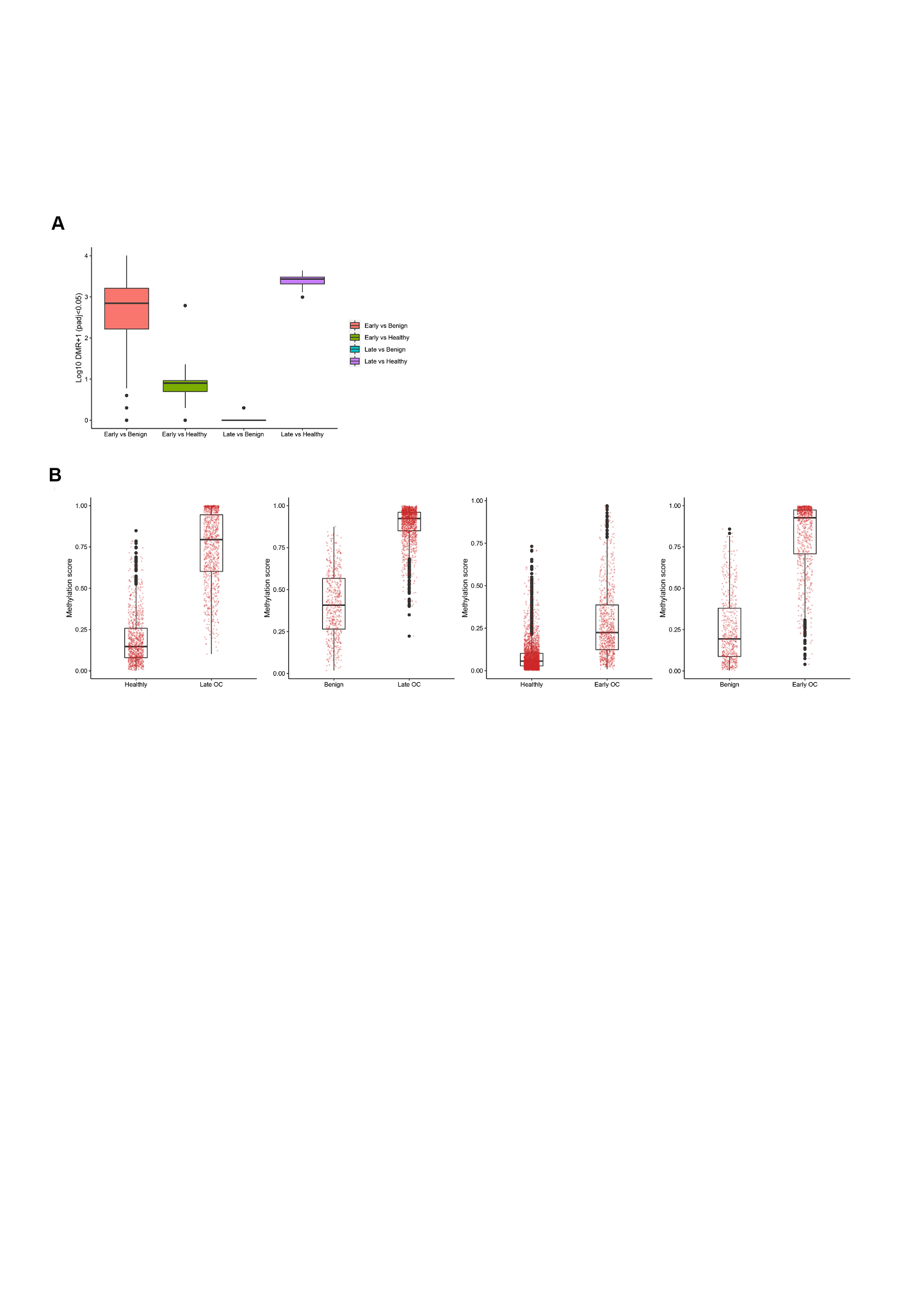


**Fig S2. DMR analysis in 100 iterations of group comparison, corresponding to Figure2.** A. Number of DMRs in 100 iterations for four group DMR analysis. Y axis represents the number of DMR+1 scaled with log10 for each iteration of four group, using the threshold of DMR with DESeq2 adjusted p value less than 0.05. B. Methylation score of the test set in 100 iterations, late-stage OC versus healthy group, late-stage OC versus benign group, early-stage OC versus healthy group, and early-stage OC versus benign group.


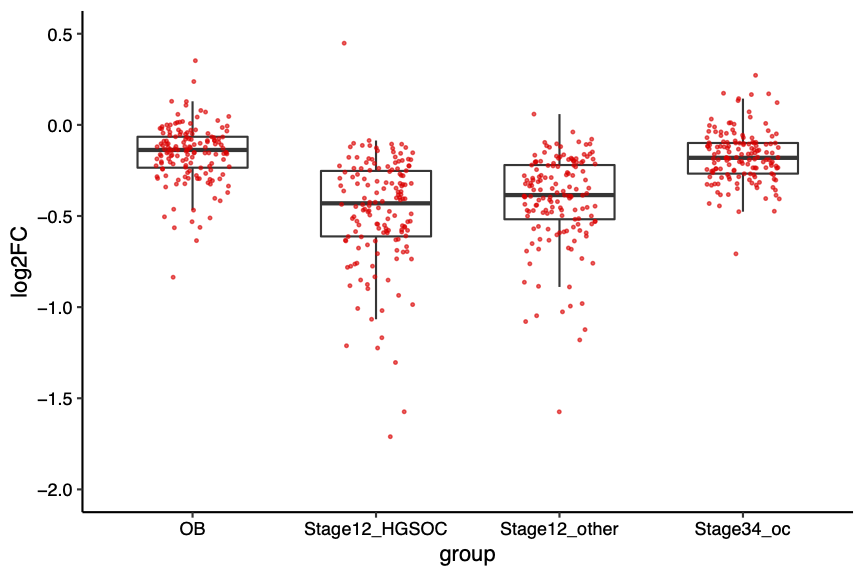


**Fig. S3 Differential methylation levels of top150 hypo DMR early to healthy DMRs in Benign, early-stage HGSOC, early-stage non-HGSOC and late-stage samples**. Each point represents one hypo DMR region. The hypomethylation levels show no discrimination between two subtypes of early-stage samples.
